# Urinary Tract Infections Caused by Uropathogenic *Escherichia coli* Strains—New Strategies for an Old Pathogen

**DOI:** 10.3390/microorganisms10071425

**Published:** 2022-07-14

**Authors:** Carlo Zagaglia, Maria Grazia Ammendolia, Linda Maurizi, Mauro Nicoletti, Catia Longhi

**Affiliations:** 1 Department of Public Health and Infectious Diseases, Microbiology Section, “Sapienza” University of Rome, 00185 Rome, Italy; carlo.zagaglia@uniroma1.it (C.Z.); linda.maurizi@uniroma1.it (L.M.); 2 National Center of Innovative Technologies in Public Health, National Institute of Health, 00185 Rome, Italy; 3 Department of Experimental Sciences and Clinics, “G. D’Annunzio” University, 66100 Chieti, Italy; mauro.nicoletti@uniroma1.it

**Keywords:** uropathogenic *E. coli*, urinary tract infections, antibiotic resistance, new therapeutic strategies

## Abstract

Urinary tract infections (UTIs) are among the most common infections worldwide. Uropathogenic *Escherichia coli* (UPECs) are the main causative agent of UTIs. UPECs initially colonize the human host adhering to the bladder epithelium. Adhesion is followed by the bacterial invasion of urothelial epithelial cells where they can replicate to form compact aggregates of intracellular bacteria with biofilm-like properties. UPEC strains may persist within epithelial urothelial cells, thus acting as quiescent intracellular bacterial reservoirs (QIRs). It has been proposed that host cell invasion may facilitate both the establishment and persistence of UPECs within the human urinary tract. UPEC strains express a variety of virulence factors including fimbrial and afimbrial adhesins, invasins, iron-acquisition systems, and toxins, which cooperate to the establishment of long lasting infections. An increasing resistance rate relative to the antibiotics recommended by current guidelines for the treatment of UTIs and an increasing number of multidrug resistant UPEC isolates were observed. In order to ameliorate the cure rate and improve the outcomes of patients, appropriate therapy founded on new strategies, as alternative to antibiotics, needs to be explored. Here, we take a snapshot of the current knowledge of coordinated efforts to develop innovative anti-infective strategies to control the diffusion of UPECs.

## 1. Introduction

Urinary tract infections (UTIs) are the most common diseases encountered in clinical practice worldwide. Despite many efforts, around 150 million people worldwide per year were still affected by UTIs, with considerable morbidity and high medical costs. Annually, over 10 million office visits, more than 2 million emergency department visits, and 100,000 hospitalizations in the United States are traced to UTIs [1,2]. UTIs are the cause of different types of diseases, including asymptomatic/symptomatic bacteriuria, acute, chronic, and recurrent infections [3,4]. Recurrences represent a major challenge in the treatment of UTI patients [5]. Children, women, elderly, diabetics, and individuals with uroliths and urinary catheters are at a higher risk of developing infections [6]. Clinically, UTIs are categorized as uncomplicated or complicated. Uncomplicated UTIs (cystitis and pyelonephritis) concern healthy patients in the absence of structural or neurological urinary tract abnormalities. Complicated UTIs are associated with factors that compromise the urinary tract or host defense, including urinary obstruction, urinary retention, immunosuppression, renal failure, pregnancy, and indwelling catheters or other drainage devices [1].

**Figure 1:**
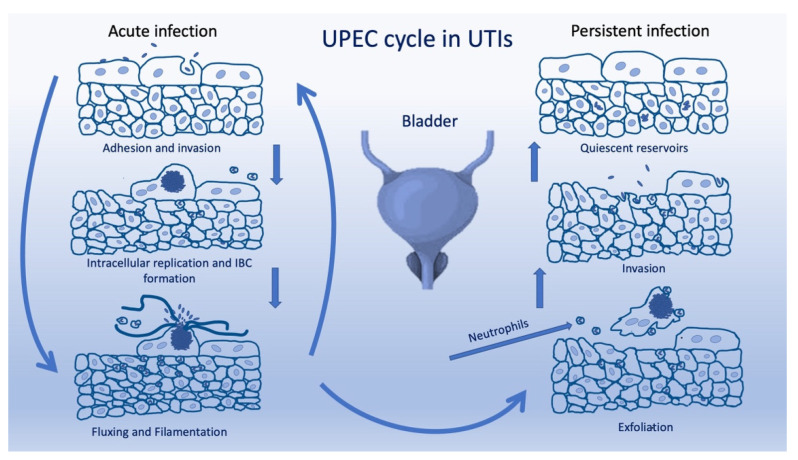
Schematic representation of the virulence mechanism of UPECs.

Uropathogenic *Escherichia coli* (UPECs), the predominant etiological agent of UTIs, account for upwards of 75% of all cases [1,6,7,8]. Given its clinical relevance, research on UPEC virulence has been pursued [9,10,11,12]. As expected, it appears that the virulence of UPECs cannot be ascribed to the carriage of a given factor, but rather to the coordinate expression of multiple genes depending on the urinary districts they colonize. In fact, although UPECs and commensal *E. coli* encode similarly, if not identically, UPECs are virulent while commensals are not. Moreover, due to the high genetic similarity, the identification of these pathogens is a difficult task such that the presence of *E. coli* strains in the urine samples of symptomatic patients is sufficient for referring to the identification of a UPEC strain [13,14]. To colonize the urinary tract, UPECs have to first avoid urinary flux (which is difficult due to adhesiveness and persistence), unstable pH levels, low-level oxygen availability, and urea [11,15]. Upon entering the urinary tract, bacteria invade and multiply within the bladder epithelial cells, forming the so-called intracellular bacterial communities (IBCs) [16]. Additionally, UPECs can form quiescent intracellular reservoirs (QIRs) that may contain non-replicating bacteria and are able to trigger reactivation by the exfoliation of superficial epithelial cells. This releases bacteria back into the bladder to cause a recurrent infection, restarting the IBC cycle (Figure 1) [1,17].

The UPEC mechanism of virulence needs the coordinated expression of a number of virulence genes. Among the virulence factors, adhesive structures (flagella, outer-membrane vesicles, pili, non-pilus adhesins, and polysaccharide capsules), outer-membrane proteins, lipopolysaccharides, and toxins, such as α-hemolysin, cytotoxic necrotizing factor 1 (CNF1), and vacuolating autotransporter cytotoxin, play a pivotal role in the ability of UPECs to colonize the hosts [9,18,19,20,21,22]. To ensure adequate levels of intracellular iron, UPECs upregulate the expression of genes involved in iron acquisition in response to iron limitation encountered within the mammalian urinary tract. UPECs capture ferric iron using siderophores, ferrous iron through iron transporters, and heme through outer-membrane receptors [7]. Biofilms allow bacteria to survive antimicrobial treatments and resist the host immune response. Biofilm formation is considered an important virulence factor that plays a relevant role in UTIs. UPECs can form biofilms on the surface of catheters, bladder walls, and within bladder epithelial cells [23,24].

## 2. Antibiotics and UPECs

As suggested by Bartoletti et al., 2016 [25], antibiotic treatment is recommended against symptomatic UTIs. Moreover, the reports of the European Association of Urology recommended fosfomycin trometamol, pivmecillinam, nitrofurantoin, and trimethoprim-sulfamethoxazole (TMP-SMZ) for the treatment of uncomplicated cystitis in all European countries [26]. Because of the relevant negative side effects, the use of fluoroquinolones as first-line antibiotics has been discouraged. Since fluoroquinolones are well absorbed by the gastrointestinal tract and can penetrate the kidney, the Infectious Diseases Society of America and European Society of Clinical Microbiology and Infectious Diseases recommend that the use of oral fluoroquinolones should be limited to the treatment of acute pyelonephritis and complicated UTIs [27]. Moreover, recently, it has been reported that fluoroquinolones are still in use for lower UTI management [28]. In the case of recurrent UTIs, a prophylaxis schedule with the administration of ‘‘booster’’ cycles of antibiotics for five days per month are used frequently but with conflicting results [25].

UPECs persist in the urinary tract in different situations, such as IBCs or QIRs, and the ability to form biofilms on biological surfaces is a characteristic that may prevent bacteria eradication during antibiotic treatments [1,29]. Using a suitable in vitro model, it has been found that some cephalosporins, amikacin, and ciprofloxacin at concentrations similar to those achieved in human urine are able to reduce UPEC-produced biofilm [30]. Different authors demonstrated that UPEC IBCs can persist despite treatment with multiple antibiotics [31,32]. Recently, a human bladder-chip model was developed for the study of the dynamic role for IBCs as harbors of bacterial persistence, with significant consequences for the non-compliance with antibiotic regimens [33]. The effect of novel antibiotics as well as of new antibiotic combinations has also been studied. A pilot study provided a first suggestion that the intravesical instillation of antibiotics may reduce the frequency of UTIs in patients with neurogenic lower urinary tract dysfunction using intermittent catheterization [34]. It has been recently reported that non-antibiotic prophylactic treatment with methenamine hippurate might be appropriate for women with a history of recurrent episodes of urinary tract infections [35]. Tazobactam-ceftolozane is a novel antibiotic therapy that has been suggested as effective in the treatment of complicated UTIs and uncomplicated pyelonephritis [36]. Prophylactic antibiotic therapy is the current standard of care to prevent UTIs in many worldwide guidelines. However, European guidelines advise prudent antibiotic prescriptions to reduce antimicrobial resistance. Antibiotic treatment is becoming increasingly challenging as multidrug resistance expands among UPEC strains worldwide [37,38]. The widespread use of fluoroquinolones, especially ciprofloxacin, in the outpatients with UTIs is the cause of a continuous increase in resistance to these drugs [39,40]. An increased resistance to trimethoprim-sulfamethoxazole, which is widely used as the first-line antimicrobial in the treatment of uncomplicated UTIs, was reported in many countries [38,41,42,43]. Some authors, describing antibiotic resistance patterns of the five most frequent causative uropathogens in a Department of Urology of a tertiary referral center in Central Europe over a period of nine years, detailed *E. coli* resistance to most antimicrobials exceeding 30% in the case of ampicillin, fluoroquinolones, and cotrimoxazole and being above 10% for amoxicillin/clavulanate, piperacillin/tazobactam, cefuroxime, and cefepime [44]. Recently, a study validated the potent inhibitors of bacterial stress protein UspA functions and indicated their potential as alternative therapeutics to combat the multidrug resistant uropathogenic *E. coli* [45].

UPEC isolates, resistant to all or nearly all antibiotics currently in use in clinical practice together with the scarcity of new antibiotics, are limiting the options for effective antibiotic therapy. Moreover, since resistance to new generation antibiotics is also emerging worldwide, coordinated efforts are greatly needed to develop innovative anti-infective strategies to control the diffusion of these highly resistant pathogens.

## 3. Natural Products Used in the UTI Treatment

**Figure 2:**
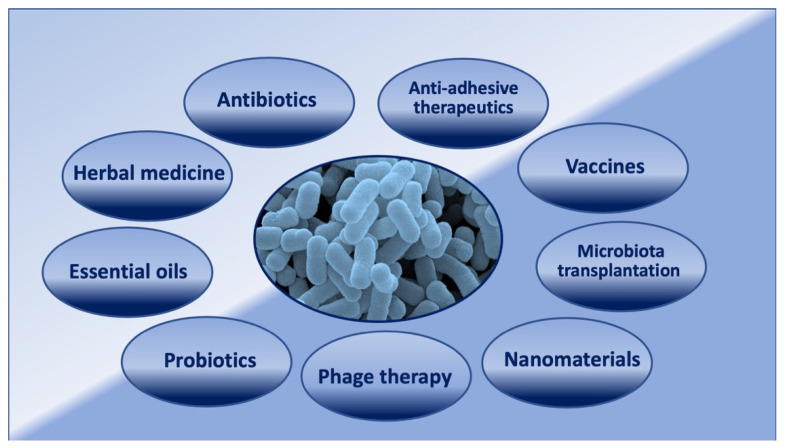
Current and new strategies used to counteract UTIs.

Natural products have always been used in the treatment and prevention of chronic and recurrent UTIs (Figure 2) [46].

Medicinal plants have always been used to cure and/or prevent UTIs. It has been calculated that, of all known and classified terrestrial plants, more than 10,000 plants are used for medical purposes [47]. For the treatment of recurrent UTIs, cranberry is the most used. Berberine and uva ursina are also prescribed for acute UTIs [48]. Berberine, a quaternary ammonium salt from the protoberberine group of alkaloids found in the plants of the family of *Berberidaceae*, was able to decrease the adhesive and invasive UPEC ability [49]. Moreover, the use of cranberries (*Vaccinium macrocarpon*), which are rich in flavonoids, flavonols, phenolic acids, and benzoates, has been extensively recommended for UTIs. The protective effect is probably due to the capacity of cranberry polyphenols to act as antiadhesive agents in preventing or inhibiting the adherence of pathogens to uroepithelial cell receptors [50]. In fact, recent studies highlight that the activity of cranberry extracts does not derive from a single compound but from a mixture of unglycosylated flavones that exert a strong anti-adhesive activity, already found in vitro in T24 bladder cells. In addition to the activity on the UPEC outer membranes, the cranberry extract also stimulates the secretion of the Tamm–Horsfall protein (THP) in the kidney. This is a strongly mannosylated glycoprotein that binds the domain of FimH, deputy to the recognition of mannose, preventing the adhesion of the bacteria to the host cells [51]. Despite the worldwide use and the properties described, EFSA reports that the evidence on consumption of proanthocyanidins from cranberry fruit for defense against bacterial pathogens in the lower urinary tract was considered insufficient to establish a cause-and-effect relationship [52]. In addition, the FDA considers that there is limited scientific evidence supporting the hypothesis that the consumption of a cranberry juice beverage or a cranberry dietary supplement could reduce the recurrent UTI risk in healthy women [53]. 

Another approach to treating UTIs is the use of natural diuretics that help in flushing out probable threats, such as *Solidago* spp. (goldenrod) herb, *Levisticum officinale* (lovage) root, *Petroselinum crispus* (parsley) fruit, and *Urtica dioica* (stinging nettle) [54]. Chinese herbal medicine has been successfully used for treating the symptoms of UTIs for over 2000 years. The combination of this method with antibiotics can alleviate the symptoms of UTIs and reduce the possibility of relapses from 30% (when antibiotics were used alone) to 4.4% [55]. The antibacterial properties and effects of the compound dictamnine, extracted from the traditional Chinese medicine *Cortex Dictamni*, on the bacterial morphology, cell adhesion, and invasion of UPECs were recently demonstrated [56]. Among the various UPEC virulence factors, those involved in the formation of the biofilm attracted particular attention. Biofilm formation can be considered a determining factor for the persistence of bacteria in the genitourinary tract and the failure of conventional therapy. A good strategy to overcome this issue is the use of essential oils (EOs) from natural plants. Several authors have demonstrated the antimicrobial and antibiofilm activity of various EOs such as oregano, thyme, and cinnamon. In addition, it has been shown that *Betula pendula* EO affected the biofilm formation by UPEC. *Origanum majorana*, *Thymus zygis*, and *Rosmarinus officinalis* EOs had important antibacterial activities against UPECs [57]. *Satureja montana* EO exerted antibacterial and antibiofilm activities and showed synergistic interactions with gentamicin against both reference and clinical *E. coli* bacterial strains [58]. *Coriandrum sativum* EO was also able to inhibit the growth and produce bacterial structural modifications of multidrug-resistant UPEC strains [59]. Furthermore, promising medicinal plants such as *Pomegranate granatum* or *Aronia melanocarpa* showed antibacterial activities against antibiotic-resistant *E. coli* strains [60].

## 4. Novel Strategies in the Prevention and Treatment of UTIs

Due to the increasing rate of antibiotic-resistant strains, new therapeutic approaches such as vaccines, receptor analogues, phage therapy, and others are considered against UTIs (Figure 2) [61,62].

### 4.1. Vaccination

Studies conducted in animal models suggested that vaccination could be promising in reducing the occurrence and severity of UTIs. Vaccines that target relevant UTI virulence factors, such as pili and flagella, appear to be the more promising. Vaccination with FimH adhesin was effective in mouse infection models and non-human primates [63]. Accordingly, after a study addressed the evaluation of IgG responses to immunization with the FimCH chaperone–adhesin complex, the FDA approved the compassionate administration of the FimCH vaccine for UTI patients who no longer respond to the standard of care [4]. Moreover, in a mouse model of chronic cystitis, vaccination with the N-terminal adhesive domain of FmlH strongly reduced the bacterial load. Moreover, it has been shown that the inoculum of truncated flagellin (FliC) derived from entero-aggregative *E. coli* is highly protective in a mouse model of immunization [64]. However, not only are pili and flagella effective immunogens in active vaccination towards UTI pathogens, outer-membrane iron-acquisition proteins, including siderophores and heme receptors expressed during bacterial *E. coli* UTIs, could represent structures for vaccine preparation [65].

### 4.2. Probiotics

Although probiotics are commonly used for the gastrointestinal tract, their usefulness has recently been extended to the urinary tract. To limit the insurgence of symptomatic UTIs, the production of biofilm and the ability to outcompete UPEC strains for growth in urine are considered relevant. *E. coli* strain 83972, a prototype of *E. coli* associated with asymptomatic bacteriuria isolated from the urine of a woman who had carried it for at least 3 years without any sign or symptoms of UTIs, has been shown to protect patients from UTIs. The molecular characterization of the *E. coli* 83972 revealed that it lacks defined O and K surface antigens and carries adhesins homologous to Fim, Pap, Uca, and Foc [66]. *E. coli* 83972 was endorsed by the European Association of Urology Guidelines in 2015. The ability of this strain to colonize the urinary tract for prolonged time periods and protect patients from symptomatic UTIs has been associated with biofilm production, rapid growth in urine, and no expression of flagella or functional P pili or type 1 pili. Others studies identified *E. coli* strains with a low potential to cause infections but with high fitness and competitiveness in urine for preventive and therapeutic bladder colonization [66,67,68]. Although the competitiveness of these strains against various uropathogenic bacterial isolates seems to be promising in the prevention of UTIs, some aspects have yet been elucidated.

It has been suggested that regulation of the gastrointestinal and vaginal flora with probiotics can avoid genitourinary infections. Probiotics containing *Lactobacillus* species are considered a good clinical tool in the prevention of UTIs [69,70].

With the increasing concern of antibiotic-resistant bacteria, engineering probiotics represent a very promising approach with high specificity and without damaging host microbiota. Different probiotics have been engineered to target a wide range of diseases, with some demonstrating high efficacy [71]. It has been hypothesized that bioengineering *Lactobacillus*, as host expression systems for urobiome-derived bacteriocins, can provide enhanced antimicrobial activity, improved yield, and can make bacteriocins more responsive to purification [72].

### 4.3. Anti-Adhesive Therapeutics

Uropathogens use various strategies to colonize host tissues and cause diseases. Novel approaches have been made to counteract bacterial adhesion. UPECs and other Gram-negative bacteria mediate colonization by pili to sugar moieties, and they can be inhibited by molecules that compete with the specific receptors. In particular, d-mannose represents the main anti-virulence therapeutic strategies for the treatment of UTIs [73,74]. The structural similarity between d-mannose and urothelial-mannosylated receptors makes d-mannose a strong inhibitor of UPEC adhesivity in the urinary tract epithelium. D-mannose is rapidly absorbed and excreted by the urinary tract where it saturates bacterial FimH, preventing its binding to the urothelial cells and facilitating the clearance of bacteria by the flow of urine [75]. D-mannose does not affect neither bacterial viability, shape, or motility and shows no interference with antibiotics therapy. Distinct from the antibiotics, this sugar did not select FimH variants that can modify bacterial adhesion after d-mannose removal [76,77,78,79]. In vivo experiments confirm that the oral administration of mannosides in mice selectively removed UPECs from urinary and gastrointestinal habitats. Distinct from antibiotics, native microbiota was not altered by mannosides in murine models of acute cystitis [73].

### 4.4. Phage Therapy

Another promising alternative to antibiotic treatment is phage therapy, which is based on the ability of phages to enter and lyse bacteria. This potential therapeutic tool shows advantages compared to the routine treatments because it retains antimicrobial activity at the site of infection with minimal influence on microbiota. Moreover, phages can be used to eliminate almost every pathogen because of their versatility and specificity. Single naturally occurring lytic phages or a mixture of different phages as well as phage combination with appropriate antibiotics or disinfectants could be innovative and constitute promising therapeutic alternative [80,81,82]. Phage mixture preparations showed potential activity in the treatment of UPEC biofilm present on the medical devices or IBCs [83]. Engineered or genetically modified phages were also developed by using genetic methods, genetic engineering, and other technologies. These strategies provided phages as a source of antibacterial agents or carriers for the delivery of therapeutic genes and drugs [80].

However, although phage therapy is a very promising and safe clinical approach for the treatment of UTIs, the number of clinical trials performed is low [83]. Nevertheless, available data regarding phage therapy of UTIs, as well as diarrheagenic UPEC, EPEC, ETEC, and *P. aeruginosa*, have not shown collateral negative effects so far, confirming the efficacy, utility and safety of phage mixtures. Overall, there is a convinced belief that, in the future, phage therapy will be a valuable alternative tool to commonly used antibiotics against UPECs [84].

### 4.5. Microbiota Transplantation

Due to the success of fecal microbiota transplantation (FMT) in the treatment of *Clostridioides difficile* infection, the potential use of FMT in treating other infectious diseases has gained interest as an alternative strategy to antibiotics [85]. The modification of the urobiome in the prevention of UTIs has been suggested. Recently, FMT used to restore a healthy microbial composition into the intestinal lumen of patients has been employed to repopulate healthy bacterial commensals in the urobiome [86,87]. FMT has also been reported to be useful for the treatment of recurrent UTIs [86,87,88,89]; but, despite the excitement due to its therapeutic potential, the evidence in this area remains at an initial standpoint.

### 4.6. Nanomaterials

Emerging promising tools for UTI treatment include the nanomaterial-based antimicrobials that provide several advantages compared to conventional antimicrobials. They have better stability over time together with improved drug release and higher therapeutic efficacy. Compared with conventional therapy, drug delivery by nanomaterials led to an enhanced specificity in targeting cells, higher solubilization of hydrophobic drugs, better bioavailability, a drug release that could to be controlled, the possible application of a synergistic combinatorial chemistry, and enhancement in drug delivery. Nanoparticles (NPs) with antimicrobial activity, based on metals such as gold (Au), silver (Ag), titanium (Ti), or metal-based oxides, have been extensively investigated [90]. NPs with antimicrobial properties themselves or the ability to increase antibiotic efficiency are referred to as nanoantibiotics [91]. Nanoantibiotics kill pathogens through multiple mechanisms such as photothermolysis, reacting oxygen species (ROS) generation, by interfering with enzymatic activities and DNA synthesis, and damaging cell wall and cell components [92]. Peculiar features of nanoparticles (small size, surface charge, shape, and high surface area) render these nanomaterials the most suitable nanocarriers for a better cellular uptake of antimicrobials. Regarding UTIs, several metallic NPs have been employed to obtain an efficient delivery of antimicrobials [93]. Ag, copper and zinc NPs showed promising antibacterial activity and the best performance compared to free antibiotics against planktonic or biofilm forms of *E. coli* [94,95,96]. Moreover, polymeric NPs have demonstrated high performances as nanocarriers because of an efficient loading of antimicrobials. To inhibit the adhesion of pathogens to the catheters, a nanoformulation based on norfloxacin, Ag NPs, and polylactic-co-glycolic acid (PLGA)-based polymers has been developed [97]. This nanocarrier, after degradation in an aqueous environment and alkali production from urea, showed significantly high anti-adhesive and antimicrobial properties.

Polymeric and hybrid polymer/metallic NPs developed for the intravesical therapy in bladder cancer were also tested for UTI treatment. Two polymeric NPs based on PLGA 2300 and PLGA 503H, surface tailored with wheat germ agglutinin, have shown significantly enhanced adhesion to human uroepithelial cells when also trimethoprim-loaded [98]. Cranberry proanthocyanidin-chitosan NPs have been investigated against *E. coli* colonization, revealing a good proanthocyanidin protection from oxidation and reduced bacterial cell invasion [99]. A biodegradable and antibacterial stent has been made by Au and Ag NPs embedded in a fiber membrane of PGA/PGLA NPs. The exfoliation of the stent surface, by a gradual degradation of PGA/PGLA, provided a rapid killing of *Staphylococcus aureus* and *E. coli* [100]. Other hybrid NPs based on polyvinylpyrrolidone-coated Ag NPs, tested for antimicrobial properties against *E. coli*, showed a higher potency compared with the uncoated NPs [101].

Carbon-based nanomaterials also were evaluated in UTI therapy. Lu et al., 2019, took advantage of graphene, carbon nanotubes, and nanodiamonds in biofilm formation prevention on the catheter’s surface [102]. These nanomaterials also showed the ability to release drugs in the target sites [103,104,105]. Nanoparticles based on dendrimers were also investigated. Dendrimers are artificial macromolecules with an increasing role in drug delivery. They had the ability to bind FimH type-1 lectins present at the tip of bacterial fimbriae, showing high potency, bioadhesion, and antibiofilm properties against *E. coli* [106]. To avoid microbe adhesion to the surface of silicone catheters, Zhu et al., 2017, generated mannoside-decorated dendrimers to increase the formation of a protective coating [107]. Nanoemulsion-based gel formulation for *Green tea* catechins and cranberry powder for intravaginal delivery was explored as innovative therapy for UTIs [108]. This nanosystem showed enhanced antibacterial activity and can be transported trans-vaginally from the vagina to the urinary tract by systemic circulation.

## 5. Concluding Remarks

An increasing resistance rate relative to the antibiotics was evidenced by current guidelines for the treatment of UTIs. Multidrug and pandrug resistance in UPEC isolates was observed. The need for a rationally designed and developed alternative treatment is, therefore, increasing. Among these, plant-based options could represent appealing choices because they are cost-effective, readily available, and reduce antimicrobial resistance hazards. However, further studies must be conducted to reveal the chemical composition linked to pharmaceutical activities and their mechanisms. Therefore, the development of preventive vaccines represents a fascinating tool. The vaccine, with whole or lysed fractions of inactivated bacteria, was suggested as effective to generate protective immunity against UTIs. Probiotics are also considered because of their ability to reduce UTI risks and vaginal infections. Phage therapy appears to be one of the most promising alternatives to fight various pathogens, including uropathogens that are resistant to commonly available antibiotics. Finally, the emerging role of nanotechnology may open up new avenues for therapeutic interventions because of the intrinsic properties of nanomaterials and their ability to target intracellular reservoirs. The combination of conventional and innovative therapies to target the interplay between intracellular and extracellular bacterial survival pathway would be desirable. Altogether, the therapeutic options highlighted in this perspective appear to be promising for the prevention of antimicrobial resistance and UTI recurrence, even though they still require considerable efforts in order to be adopted into clinical practice.
